# 

**Published:** 1857-12

**Authors:** 


### CONTENTS.

ORIGINAL ESSAYS AND COMMUNICATIONS.

Introductory Lecture to the General Course of Mechanical Dentistry. By J. Richardson, D. D. S..... 121
Filling Teeth. By J. Taft..... 137
Third Annual Meeting of the American Dental Convention..... 145
Interesting Case of Hemicrania. By J. Levison, D. D. S., &c..... 156
Cheoplasty..... 159
Boston Meeting..... 163
Little Troubles with a Plate Tooth. By A. Berry, D. D, S..... 168
Functions of the Nervous System,..... 168

PROCEEDINGS OF SOCIETIES.

Dental Convention of Northern Ohio..... 171
Miscellaneous..... 204
Editorial..... 229

